# Immobilization rapidly induces muscle insulin resistance together with the activation of MAPKs (JNK and p38) and impairment of AS160 phosphorylation

**DOI:** 10.14814/phy2.12876

**Published:** 2016-08-01

**Authors:** Emi Kawamoto, Keiichi Koshinaka, Tatsuhiko Yoshimura, Hiroyuki Masuda, Kentaro Kawanaka

**Affiliations:** ^1^ Department of Health and NutritionNiigata University of Health and WelfareNiigataJapan; ^2^ Department of Materials EngineeringNagaoka National College of TechnologyNagaokaJapan; ^3^ Faculty of Sports and Health ScienceFukuoka UniversityFukuokaJapan

**Keywords:** AS160, c‐Jun NH2‐terminal kinase, hindlimb immobilization, insulin resistance, p38 MAPK, serine palmitoyl transferase‐2, skeletal muscle

## Abstract

Acute short‐duration physical inactivity induces the development of insulin resistance for glucose uptake in skeletal muscle. We examined the possibility that inactivity rapidly induces muscle insulin resistance via the excessive activation of proinflammatory/stress pathways including those of IKK/I*κ*B/NF‐*κ*B, JNK, and p38 MAPK. We also examined the other possibility that inactivity‐induced rapid development of insulin resistance is associated with reduced phosphorylation of AS160, the most distal insulin‐signaling protein that have been linked to the regulation of glucose uptake. Male Wistar rats were subjected to unilateral hindlimb immobilization for 6 h. At the end of the immobilization, the soleus muscles from both immobilized and contralateral non‐immobilized hindlimbs were dissected out. Immobilization decreased insulin‐stimulated 2‐deoxyglucose uptake in rat soleus muscle within 6 h. This rapid development of insulin resistance was accompanied by elevated phosphorylation of both JNK and p38 (commonly used indicator of JNK and p38 pathway activity, respectively). In addition, the abundance of SPT2, a rate‐limiting enzyme regulating ceramide biosynthesis, was increased in immobilized muscle. Immobilization did not alter the abundance of I*κ*B*α* (commonly used indicator of IKK/I*κ*B/NF‐*κ*B pathway activity). The basal phosphorylation of AS160 at Thr642 and Ser588 was decreased together with the development of insulin resistance. These results suggest the possibility that inactivity‐induced rapid development of insulin resistance in immobilized muscle is related to enhanced activation of JNK and/or p38. Elevated ceramide biosynthesis pathway may contribute to this activation. Our results also indicate that decreased basal phosphorylation of AS160 may be involved in inactivity‐induced insulin resistance.

## Introduction

Chronic muscle disuse – produced by the condition of prolonged physical inactivity associated with bed rest, denervation, or limb immobilization – induces insulin resistance in human and rodent skeletal muscle (Henriksen et al. 1991; Didyk et al. 1994; Blakemore et al. 1996; Bienso et al. 2012; Friedrichsen et al. 2012; Mortensen et al. 2014). Surprisingly, it was previously confirmed that hindlimb immobilization induces insulin resistance for glucose uptake in mouse soleus muscle within 6 h (Nicholson et al. 1984), suggesting that insulin resistance is induced very rapidly in response to a bout of short‐duration physical inactivity. However, the precise cellular mechanism by which inactivity rapidly induces insulin resistance in skeletal muscle remains to be identified. We examined the two possibilities regarding the mechanisms of the inactivity‐induced rapid development of muscle insulin resistance.

Our first hypothesis regarding the mechanism by which a bout of physical inactivity rapidly induces muscle insulin resistance is the activation of proinflammatory/stress pathways that restrain insulin signaling and insulin‐stimulated glucose uptake. Of these pathways, the inhibitor of *κ*B kinase (IKK)/I*κ*B/nuclear factor‐*κ*B (NF‐*κ*B) and mitogen‐activated protein kinase (MAPK) pathways, such as c‐Jun NH2‐terminal kinase (JNK), p38 MAPK, and extracellular signal‐regulated kinase (ERK) are best described. For example, it has been postulated that excessive activation of the IKK/I*κ*B/NF‐*κ*B pathway may be an important molecular mechanism responsible for skeletal muscle insulin resistance (Kim et al. 2001; Yuan et al. 2001; Itani et al. 2002; Bhatt et al. 2006). Moreover, JNK activity was also shown to be abnormally elevated in insulin‐resistant skeletal muscle of obese mice (Hirosumi et al. 2002; Sabio et al. 2010). Other studies demonstrated that the activation of p38 MAPK plays a critical role in the development of insulin resistance (Blair et al. 1999; Koistinen et al. 2003; De Alvaro et al. 2004; Diamond‐Stanic et al. 2011). There are also multiple lines of evidence linking greater stimulation of ERK to insulin resistance (Csibi et al. 2010; Green et al. 2011; Jager et al. 2011). Moreover, since proinflammatory/stress pathways are activated within several hours in response to acute stress‐inducing stimuli (Blair et al. 1999; Urano et al. 2000; Itani et al. 2002; Bhatt et al. 2006; Diamond‐Stanic et al. 2011), it thus seems reasonable to speculate that inactivity rapidly induces muscle insulin resistance for glucose uptake via the activation of these pathways. Accordingly, our first aim was to determine whether the rapid development of insulin resistance for glucose uptake in immobilized rat soleus muscle is accompanied by the activation of IKK, JNK, p38 MAPK, and/or ERK.

Our second hypothesis regarding the mechanism of muscle insulin resistance linked to acute inactivity is the decreased phosphorylation of Rab‐GTPase‐activating protein (GAP), that is, AS160 (Akt substrate of 160 kDa; also known as TBC1D4) and its paralog TBC1D1 (tre‐2/USP6, BUB2, cdc 16 domain family member 1). In skeletal muscle, insulin binding to its receptor initiates the activation of insulin receptor substrates and phosphatidylinositol (PI) 3‐kinase, leading to phosphorylation and activation of Akt. Akt phosphorylates AS160 (Bruss et al. 2005), which is currently recognized as the most distal signaling events associated with insulin‐stimulated GLUT4 translocation and glucose uptake in skeletal muscle (Kramer et al. 2006b; Chen et al. 2011). In this context, our second aim was to determine whether the rapid development of insulin resistance for glucose uptake in immobilized rat soleus muscle is accompanied by decreased phosphorylation of AS160.

## Materials and Methods

### Materials

Antibodies against phospho‐Akt Ser^473^ (#9271), phospho‐Akt Thr^308^ (#9275), phospho‐TBC1D1 Thr^590^ (#6927), phospho‐JNK Thr^183^/Tyr^185^ (#9251), phospho‐p38 MAPK Thr^180^/Tyr^182^ (#9216), phospho‐ERK1/2 Thr^202^/Tyr^204^ (#9101), total Akt (#9272), total TBC1D1 (#4629S), total‐JNK (#9252), total‐p38 MAPK (#9212), total‐ERK1/2 (#9102), total I*κ*B*α* (#9242), and total‐acetyl CoA carboxylase (ACC, #3662) were from Cell Signaling Technology (Beverly, MA). Antibodies against phospho‐AS160 Thr^642^ (#07‐802), phospho‐ACC Ser^79^ (#07‐303), phospho‐TBC1D1 Ser^237^ (#07‐2268), and total AS160 (#07‐741) were from Millipore (Temecula, CA). Anti‐phospho‐AS160 Ser^588^ (#3028P2) was from Symansis Limited (Timaru, New Zealand). Anti‐GLUT4 antibody (#4670‐1704) was from Bio‐Rad AbD Serotec (Oxford, UK). Anti‐SPT2 antibody (ab23696) was from Abcam (Cambridge, MA). Horseradish peroxidase (HRP)‐conjugated anti‐rabbit IgG was from Biosource International (Camarillo, CA). HRP‐conjugated anti‐sheep IgG was from Millipore. HRP‐conjugated anti‐mouse IgG was from Santa Cruz Biotechnology. Enhanced chemiluminescence reagents (ECL, ECL plus, and ECL Prime) were obtained from GE Healthcare Life Sciences (Uppsala, Sweden). All other reagents were obtained from Sigma‐Aldrich (St. Louis, MO).

### Treatment of animals

This research was approved by the Animal Studies Committee of Niigata University of Health and Welfare. Three‐week‐old male Wistar rats were obtained from CLEA Japan (Tokyo). Animals were maintained in individual cages and fed a standard rodent chow diet and water ad libitum.

In Experiment 1, rats (40–50 g) were subjected to unilateral hindlimb immobilization. A plaster cast (Castlight, ALCARE Co., Tokyo) was applied to the left hindlimb of rats without anesthetization. The leg was immobilized at the plantar flexion position. After casting, rats were housed individually. Immobilization was imposed for 6 h. To ensure that the muscles from the contralateral non‐immobilized leg could be used as controls, some rats were not casted. All rats were fasted for approx. 24 h before the muscle sampling.

For the muscle sampling, the casts were removed, and the soleus muscles from both the immobilized and contralateral hindlimbs were dissected out under pentobarbital sodium anesthesia (5 mg/100 g body wt) for subsequent incubation as described below. A part of each muscle was clamp‐frozen without incubation for a subsequent western blot as described below.

In Experiment 2, rats (40–50 g) were subjected to unilateral hindlimb immobilization for 6 h. To examine the effects of recovery following cast immobilization, casts were removed without anesthetization immediately after 6 h of immobilization. Then, the rats were allowed to recover for 0 h or 6 h. At the end of the recovery period, the soleus muscles from both the immobilized and contralateral hindlimbs were dissected out under pentobarbital sodium anesthesia (5 mg/100 g body wt) for subsequent incubation as described below. A part of each muscle was clamp‐frozen without incubation for subsequent western blot analysis as described below. The muscles from the contralateral non‐immobilized leg were used as controls.

In Experiment 3, rats (40–50 g) were subjected to unilateral hindlimb immobilization for 24 h or 72 h. All rats were fasted for approx. 24 h before the muscle sampling. Soleus muscles from both the immobilized and contralateral hindlimbs were dissected out under pentobarbital sodium anesthesia (5 mg/100 g body wt) and clamp‐frozen for a subsequent western blot as described below. The muscles from the contralateral non‐immobilized leg were used as controls.

### Muscle incubation

The soleus muscles were incubated with shaking for 20 min at 30°C in 3 mL of oxygenated Krebs–Henseleit buffer (KHB) containing 40 mmol/L mannitol, 0.1% radioimmunoassay (RIA)‐grade bovine serum albumin (BSA) in the absence or presence of purified human insulin (50 or 10,000 *μ*U/mL). Flasks were gassed continuously with 95% O_2_–5% CO_2_ during incubation. After incubation, the soleus muscles were used for the measurement of 2‐deoxyglucose (2DG) uptake or were blotted, clamp‐frozen in liquid nitrogen, and then processed for a western blot analysis to measure the phosphorylation levels of Akt, AS160, and TBC1D1.

### Measurement of 2DG uptake

The rate of muscle glucose uptake was determined by measuring the 2DG uptake in isolated soleus muscle, as described (Ueyama et al. 2000; Koshinaka et al. 2008, 2009). After a 20‐min incubation as described above, soleus muscles were incubated for 20 min at 30°C in 3 mL of KHB containing 8 mmol/L 2DG, 32 mmol/L mannitol, and 0.1% BSA in the presence or absence of purified human insulin (50 or 10,000 *μ*U/mL) if present in the previous incubation. The flasks were gassed continuously with 95% O_2_–5% CO_2_ during the incubation. After the incubation, the muscles were blotted and then clamp‐frozen in liquid nitrogen. The concentration of 2‐deoxyglucose‐6‐phosphate (2DG6P) in muscles was determined as previously described (Koshinaka et al. 2008, 2009).

### Western blot analysis

Soleus muscles were homogenized in ice‐cold buffer containing 50 mmol/L HEPES (pH 7.4), 150 mmol/L NaCl, 10% glycerol, 1% Triton X‐100, 1.5 mmol/L MgCl_2_, 1 mmol/L EDTA, 10 mmol/L Na_4_P_2_O_7_, 100 mmol/L NaF, 2 mmol/L Na_3_VO_4_, 2 mmol/L PMSF, aprotinin (10 *μ*g/mL), leupeptin (10 *μ*g/mL), and pepstatin (5 *μ*g/mL) (Margolis et al. 1990). The homogenates were then rotated end‐over‐end at 4°C for 60 min and centrifuged at 4000 × *g* for 30 min at 4°C. Aliquots of the supernatants were treated with 2 × Laemmli sample buffer containing 100 mmol/L dithiothreitol.

All samples were subjected to 10% SDS‐PAGE with the exception of phospho‐AS160, total‐AS160, phospho‐TBC1D1, total‐TBC1D1, phospho‐ACC, and total‐ACC. For the measurement of phospho‐AS160, total‐AS160, phospho‐TBC1D1, total‐TBC1D1, phospho‐ACC, and total‐ACC, the samples were run on 5% SDS‐PAGE. The resolved proteins were then transferred to PVDF membranes, blocked in 5% non‐fat dry milk in Tris‐buffered saline containing 0.1% Tween 10 (TBST), pH 7.5. After blocking, the membranes were rinsed in TBST, incubated overnight with the appropriate antibody at 4°C, followed by rinsing in TBST and incubation for 120 min with HRP‐conjugated anti‐rabbit IgG, HRP‐conjugated anti‐mouse IgG, or HRP‐conjugated anti‐sheep IgG. Antibody‐bound protein was visualized by enhanced chemiluminescence (ECL, ECL plus, or ECL Prime) with the intensity of the bands being quantified using densitometry. Equal protein concentrations were loaded in each lane as also confirmed by Ponceau S staining the blot membrane.

### Statistical analysis

Data are expressed as means ± SE. When an analysis comparing more than four groups was required, a two‐way ANOVA was used to identify the source of significant variance, and a Tukey's post hoc test was used to identify the source of significant variance. When an analysis comparing two groups was required, unpaired Student's t‐test was used to determine significant differences. Differences between groups were considered significant when *P* < 0.05.

## Results

### The effects of 6‐h cast immobilization on glucose uptake and GLUT4 abundance in rat soleus muscle

**Figure 1:**
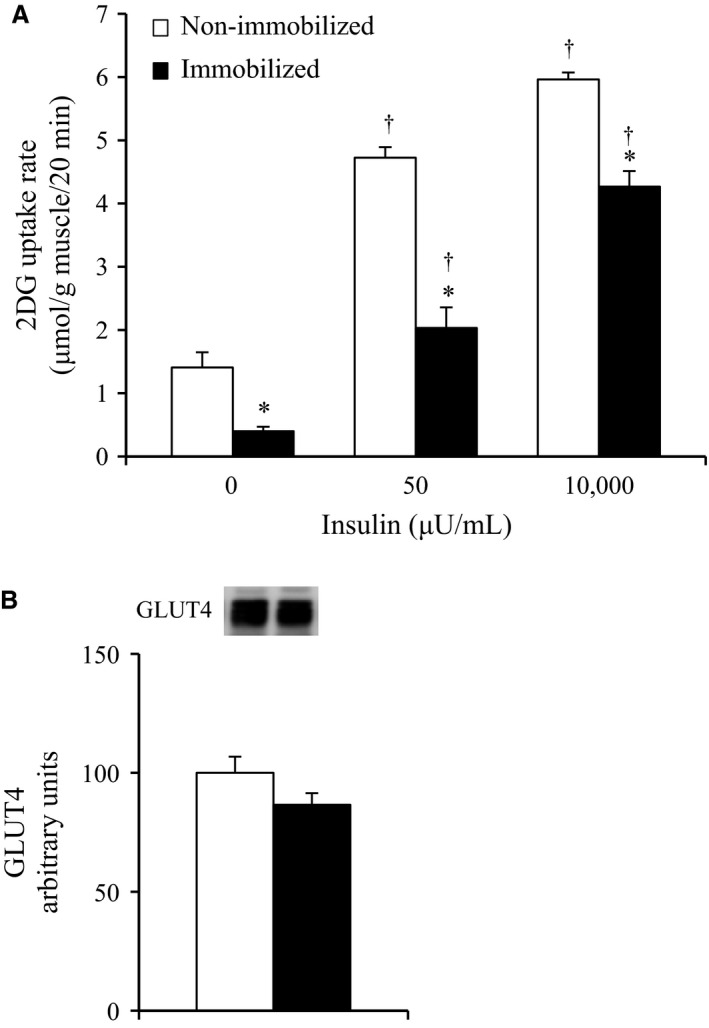
(A) Basal and insulin‐stimulated glucose uptakes in rat soleus muscle in contralateral non‐immobilized and immobilized limbs at the end of 6‐h hindlimb immobilization. Muscles were dissected out at the end of 6‐h unilateral hindlimb immobilization. All muscles were incubated in glucose‐free medium in the absence or presence (50 or 10,000 *μ*U/mL) of insulin for 20 min, followed by the measurement of 2‐deoxyglucose uptake. Values are means ± SE (*n* = 5–6). *P < 0.05 versus contralateral non‐immobilized limbs with same insulin concentration. ^†^ *P* < 0.05 versus 0 *μ*U/mL insulin. (B) Total GLUT4 protein concentration at the end of 6‐h hindlimb immobilization. Muscles were dissected out and frozen at the end of 6‐h unilateral hindlimb immobilization. Muscle lysates were separated with SDS‐PAGE, and blots were analyzed for GLUT4 protein content. Values are means ± SE (*n* = 8).

Submaximal (50 *μ*U/mL) and maximal (10,000 *μ*U/mL) doses of insulin significantly increased the 2DG uptake in both the non‐immobilized and immobilized soleus muscles, respectively (*P* < 0.05, Fig. 1A). The basal (0 *μ*U/mL), submaximal (50 *μ*U/mL), and maximal (10,000 *μ*U/mL) insulin‐stimulated 2DG uptakes were reduced in the immobilized soleus muscles by 72%, 57%, and 28%, respectively, compared to the contralateral non‐immobilized muscles after 6 h of immobilization (*P* < 0.05, Fig. 1A).

There were no significant differences in both basal (0 *μ*U/mL) and submaximal (50 *μ*U/mL) insulin‐stimulated 2DG uptake in the soleus muscles between the non‐immobilized legs of the naïve control rats without casting and the contralateral non‐immobilized legs of the rats with unilateral casting (non‐immobilized legs of naïve control rats: 1.70 ± 0.12 μmol/g tissue/20 min, *n* = 5; contralateral non‐immobilized legs of rats with unilateral casting: 1.41 ± 0.24 μmol/g tissue/20 min, *n* = 6). There was also no significant difference in the submaximal (50 *μ*U/mL) insulin‐stimulated 2DG uptake in the soleus muscles between the non‐immobilized legs of the naïve control rats without casting and the contralateral non‐immobilized legs of the rats with unilateral casting (non‐immobilized legs of naïve control rats: 4.97 ± 0.20 μmol/g tissue/20 min, *n* = 6; contralateral non‐immobilized legs of rats with unilateral casting: 4.73 ± 0.17 μmol/g tissue/20 min, *n* = 6).

There was no significant change in total GLUT4 abundance in the soleus muscles after 6‐h of immobilization (Fig. 1B).

### The effects of 6‐h cast immobilization on the phosphorylation of Akt (Ser473), Akt (Thr308), AS160 (Thr642), AS160 (Ser588), TBC1D1 (Thr590), and TBC1D1 (Ser237) in rat soleus muscle

**Figure 2:**
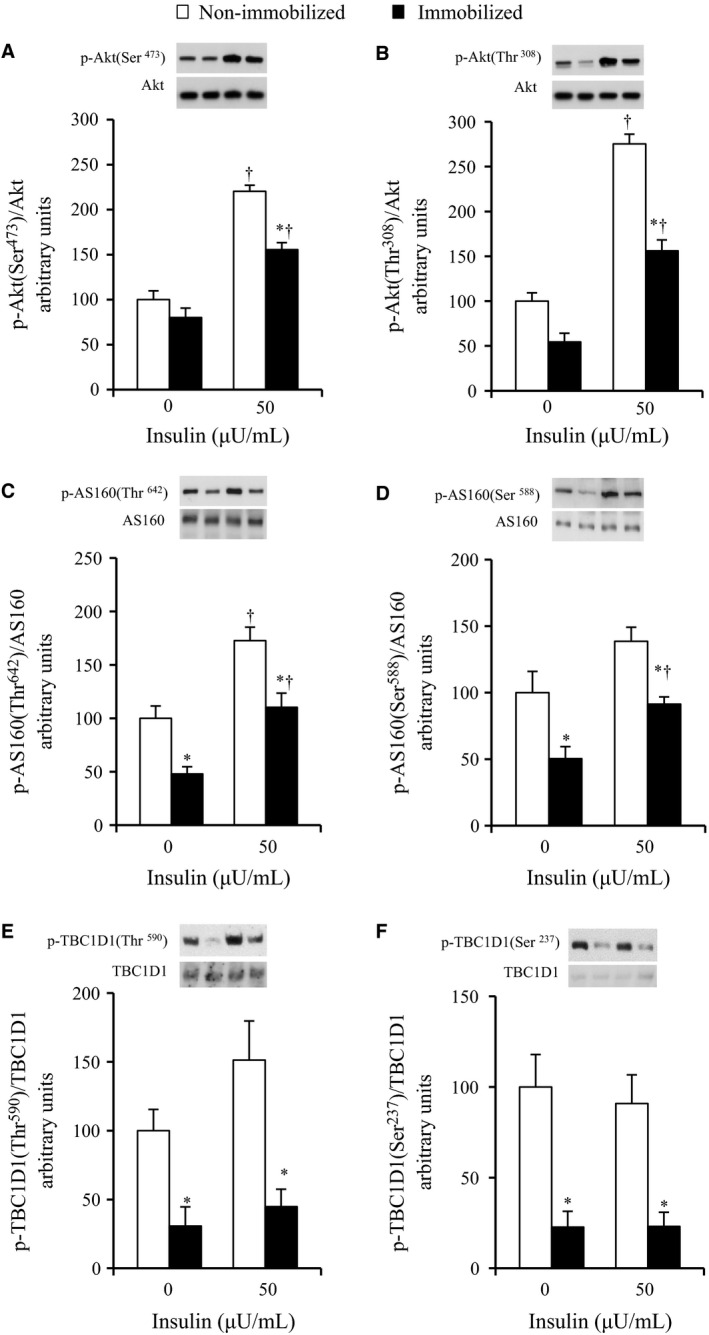
Phosphorylations of Akt, AS160, and TBC1D1 in contralateral non‐immobilized and immobilized limbs at the end of 6‐h hindlimb immobilization. Muscles were dissected out at the end of 6‐h unilateral hindlimb immobilization. All muscles were incubated in glucose‐free medium in the absence or presence (50 *μ*U/mL) of insulin for 20 min and then frozen. Muscle lysates were separated with SDS‐PAGE and blots were analyzed for phosphorylated Akt Ser473 (A), phosphorylated Akt Thr308 (B), phosphorylated AS160 Thr647 (C), phosphorylated AS160 Ser588 (D), phosphorylated TBC1D1 Thr590 (E), and phosphorylated TBC1D1 Ser237 (F). Blots were then stripped and analyzed for total abundance of each protein. (A–C, F) Values are means ± SE (*n* = 7–9). (D–E) Values are means ± SE (*n* = 6–7). *P < 0.05 versus the contralateral non‐immobilized limbs with the same insulin concentration. ^†^ *P* < 0.05 versus 0 *μ*U/mL insulin.

When expressed as a ratio of phosphorylated to total protein abundance, the Akt Ser473 and Akt Thr308 phosphorylation were increased with the submaximal (50 *μ*U/mL) insulin treatment in the muscles of both the non‐immobilized and immobilized legs (Fig. 2A and B). No significant effects of immobilization on the insulin‐independent basal phosphorylation of either Akt Ser473 and Thr308 were found, whereas the phosphorylation of Akt Ser473 with the submaximal (50 *μ*U/mL) insulin treatment was 29% lower in the muscles of the immobilized legs relative to the muscles of the contralateral non‐immobilized legs (*P* < 0.05, Fig. 2A). The insulin‐stimulated phosphorylation of Akt Thr308 was also reduced by 43% in the muscles of the immobilized legs relative to the muscles of the contralateral non‐immobilized legs (*P* < 0.05, Fig. 2B).

The phosphorylation of AS160 Thr642 was increased with submaximal (50 *μ*U/mL) insulin in the muscles of both the non‐immobilized and immobilized legs (*P* < 0.05, Fig. 2C). The basal and insulin‐stimulated phosphorylation of AS160 Thr642 were 50% and 36% lower, respectively, in the muscles of the immobilized legs compared to the muscles of the contralateral non‐immobilized legs (*P* < 0.05, Fig. 2C). The phosphorylation of AS160 Ser588 was not significantly altered with submaximal (50 *μ*U/mL) insulin in the muscles of either the non‐immobilized or immobilized legs (Fig. 2D). The basal and insulin‐stimulated phosphorylation of AS160 Ser588 were 50% and 34% lower in the muscles of the immobilized legs (*P* < 0.05, Fig. 2D).

The phosphorylation of TBC1D1 Thr590 and TBC1D1 Ser237 were not significantly altered with submaximal (50 *μ*U/mL) insulin in the muscles of either the non‐immobilized or immobilized legs (Fig. 2E and F). The basal and insulin‐stimulated phosphorylation of TBC1D1 Thr590 were 79% and 64% lower, respectively, in the muscles of the immobilized legs compared to the muscles of the contralateral non‐immobilized legs (*P* < 0.05, Fig. 2E). The basal and insulin‐stimulated phosphorylation of TBC1D1 Ser237 were 77% and 75% lower, respectively, in the muscles of the immobilized legs compared to the muscles of the contralateral non‐immobilized legs (*P* < 0.05, Fig. 2F).

### The effects of 6‐h cast immobilization on the phosphorylation of JNK, p38, and ERK in rat soleus muscle

**Figure 3:**
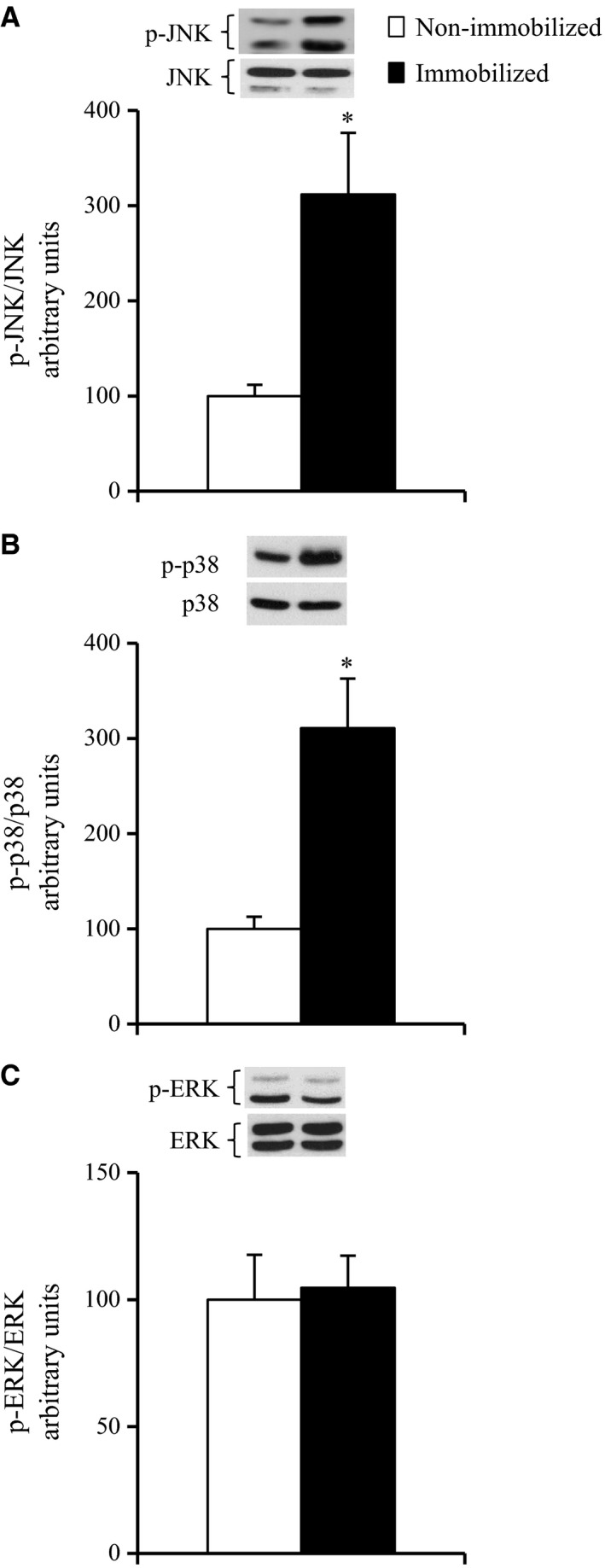
Activation of MAPK pathways in contralateral non‐immobilized and immobilized limbs at the end of 6‐h hindlimb immobilization. Muscles were dissected out and frozen at the end of 6‐h unilateral hindlimb immobilization. Muscle lysates were separated with SDS‐PAGE, and blots were analyzed for phosphorylated JNK (A), phosphorylated p38 MAPK (B), and phosphorylated ERK (C). Blots were then stripped and analyzed for total abundance of each protein. Values are means ± SE (*n* = 7–8). *P < 0.05 versus the contralateral non‐immobilized limbs.

We determined the activation of JNK, p38, and ERK, as measured by phosphorylation levels. When expressed as a ratio of phosphorylated to total protein abundance, the phosphorylation of JNK and p38 were 3.1‐fold and 3.1‐fold greater, respectively, in the muscles of the immobilized legs compared to the muscles of the contralateral non‐immobilized legs (*P* < 0.05, Fig. 3A and B). ERK phosphorylation was not significantly altered in the muscles of the immobilized legs compared to the muscles of the contralateral non‐immobilized legs (Fig. 3C).

### The effects of cast removal on glucose uptake and the phosphorylation of JNK and p38 in rat soleus muscle

**Figure 4:**
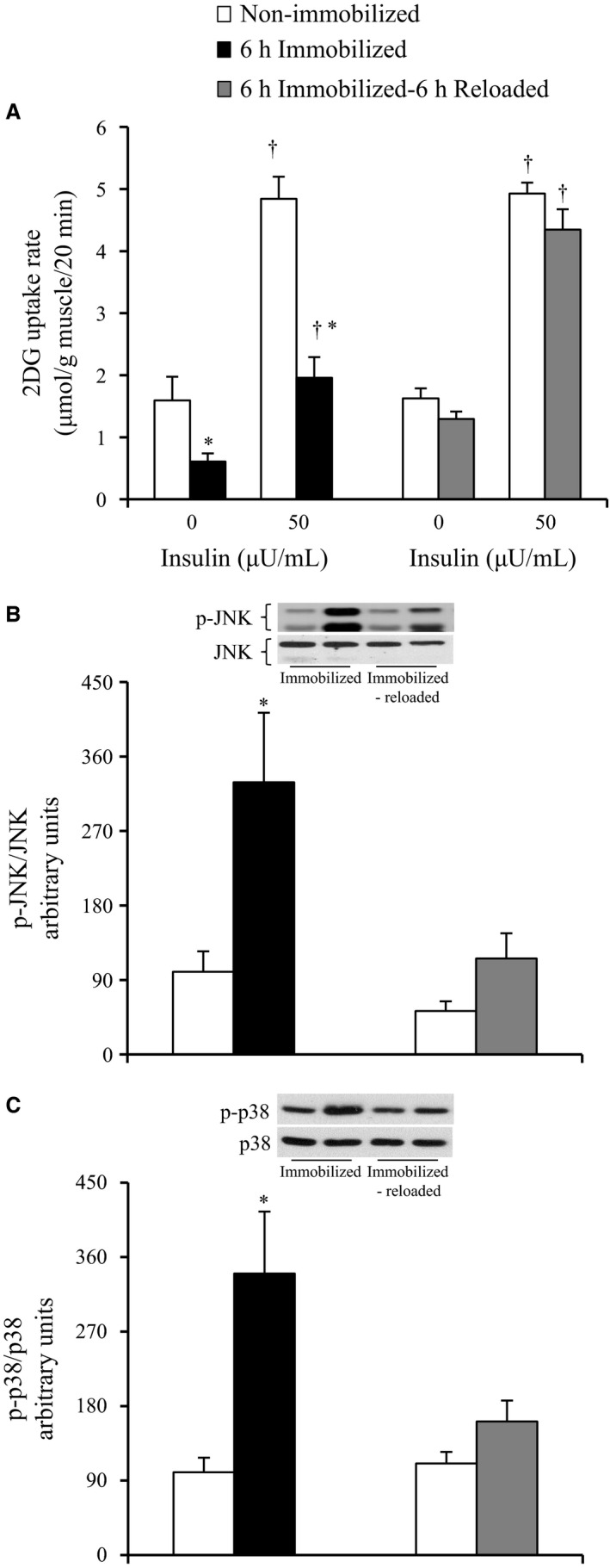
Glucose uptake rate, phospho‐JNK, and phospho‐p38 MAPK in contralateral non‐immobilized and immobilized limbs after 6‐h hindlimb immobilization + 6‐h reloading. Rats were subjected to unilateral hindlimb immobilization for 6 h. Muscles were dissected out at the end of the immobilization (6 h immobilized) or at 6 h after the cessation of immobilization (6 h immobilized − 6 h reloaded). Muscles for measuring glucose uptake were incubated in glucose‐free medium in the absence or presence (50 *μ*U/mL) of insulin for 20 min, followed by measurement of 2DG uptake (A). Muscles for measuring the phosphorylation of JNK and p38 MAPK were frozen without incubation. Muscle lysates were separated with SDS‐PAGE, and blots were analyzed for phosphorylated JNK (B) and phosphorylated p38 MAPK (C). Blots were then stripped and analyzed for total abundance of each protein. (A) Values are means ± SE (*n* = 8–9). (B,C) Values are means ± SE (*n* = 7–9). *P < 0.05 versus the contralateral non‐immobilized limbs with the same insulin concentration. ^†^ *P* < 0.05 versus 0 h after the cessation of immobilization.

Immediately after cast removal (6 h immobilized), the basal (0 *μ*U/mL) and submaximal (50 *μ*U/mL) insulin‐stimulated 2DG uptakes were reduced in the immobilized soleus muscles by 62% and 60%, respectively, compared to the contralateral non‐immobilized muscles (*P* < 0.05, Fig. 4A). In contrast, at 6‐h after cast removal (6 h immobilized − 6 h reloaded), the basal (0 *μ*U/mL) and submaximal (50 *μ*U/mL) insulin‐stimulated 2DG uptakes were not reduced in the immobilized soleus muscles (Fig. 4A).

Immediately after cast removal (6 h immobilized), JNK phosphorylation was elevated by 3.3‐fold in the muscles of the immobilized legs relative to the muscles of the contralateral non‐immobilized legs (*P* < 0.05, Fig. 4B). At 6‐h after cast removal (6 h immobilized − 6 h reloaded), no significant elevation of JNK phosphorylation was found in the muscles of the immobilized legs (Fig. 4B).

Immediately after cast removal (6 h immobilized), p38 phosphorylation was elevated by 3.4‐fold in the muscles of the immobilized legs relative to the muscles of the contralateral non‐immobilized legs (*P* < 0.05, Fig. 4C). At 6‐h after cast removal (6 h immobilized − 6 h reloaded), no significant increase in p38 phosphorylation was observed in the muscles of the immobilized legs (Fig. 4C).

### The effects of 6‐h cast immobilization on the total‐ACC abundance and phospho‐ACC in rat soleus muscle

**Figure 5:**
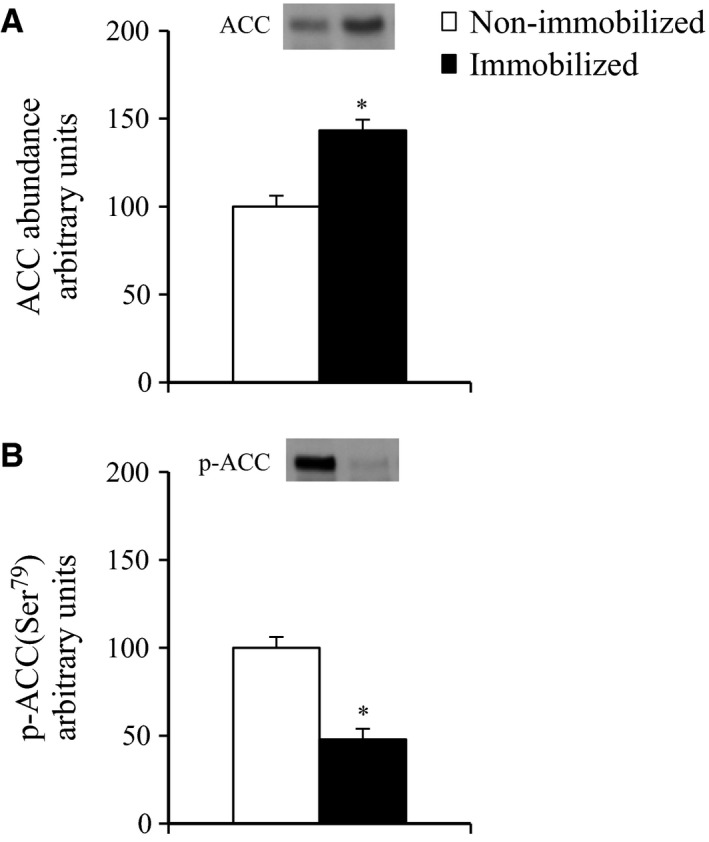
ACC abundance and ACC phosphorylation in contralateral non‐immobilized and immobilized limbs at the end of 6‐h hindlimb immobilization. Rats were subjected to unilateral hindlimb immobilization for 6 h. Muscles were dissected out and frozen at the end of the 6‐h unilateral hindlimb immobilization. Muscle lysates were separated with SDS‐PAGE, and the blots were analyzed for total‐ACC (A), and phosphorylated ACC Ser79 (B). Values are means ± SE (*n* = 7–8). *P < 0.05 versus contralateral non‐immobilized limbs.

The total ACC abundance was 43% greater (*P* < 0.05, Fig. 5A), whereas the phosphorylation of ACC Ser79 was 52% lower in the muscles of the immobilized legs compared to the muscles of the contralateral non‐immobilized legs (*P* < 0.05, Fig. 5B), indicating the increased expression and activity level of ACC in the immobilized soleus muscles, since ACC is inactivated by phosphorylation.

### The effects of 6‐h and 24‐h cast immobilization on the SPT2 abundance in rat soleus muscle

**Figure 6:**
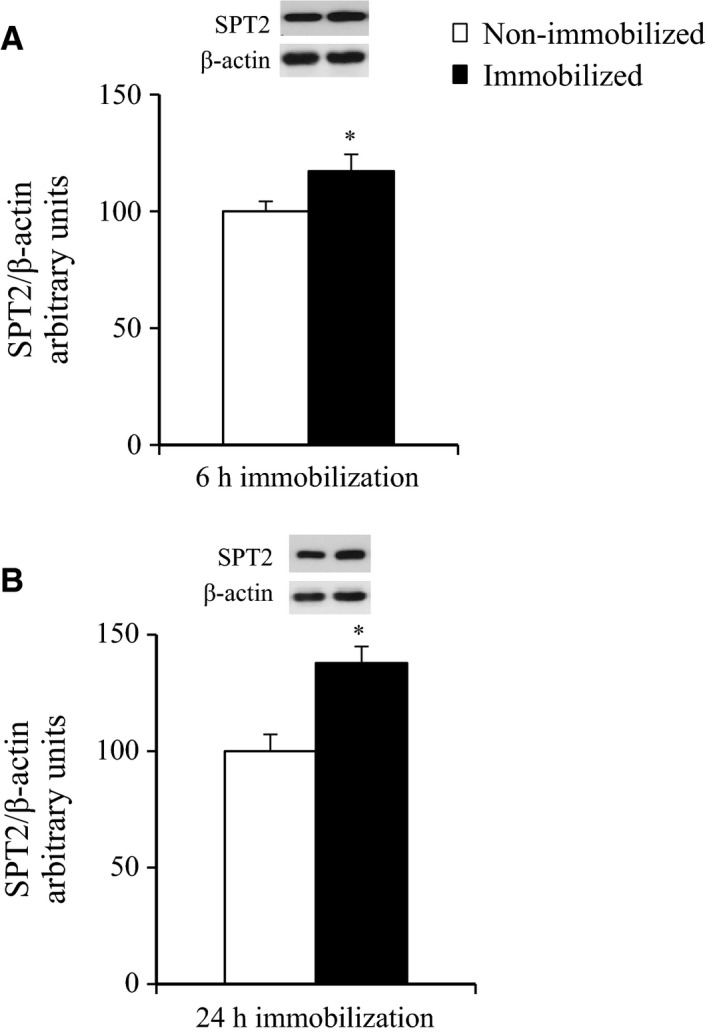
Abundance of SPT2 protein at the end of 6‐h or 24‐h hindlimb immobilization. Muscles were dissected out and frozen at the end of 6‐h (A), 24‐h (B) unilateral hindlimb immobilization. Muscle lysates were separated with SDS‐PAGE, and blots were analyzed for SPT2 protein content. Blots were then stripped and analyzed for *β*‐actin. (A) Values are means ± SE (*n* = 7). (B) Values are means ± SE (*n* = 6). *P < 0.05 versus contralateral non‐immobilized limbs.

We estimated the abundance of serine palmitoyltransferase 2 (SPT2), which is the rate‐limiting enzyme regulating ceramide biosynthesis from palmitate. At the end of the 6‐h and 24‐h immobilization, the abundance of SPT2 was increased in the immobilized soleus muscles by 17% and 38%, compared to the contralateral non‐immobilized muscles (*P* < 0.05, Fig. 6A and B),

### The effects of 6‐h, 24‐h, and 72‐h cast immobilization on the I*κ*B*α* abundance in rat soleus muscle

**Figure 7:**
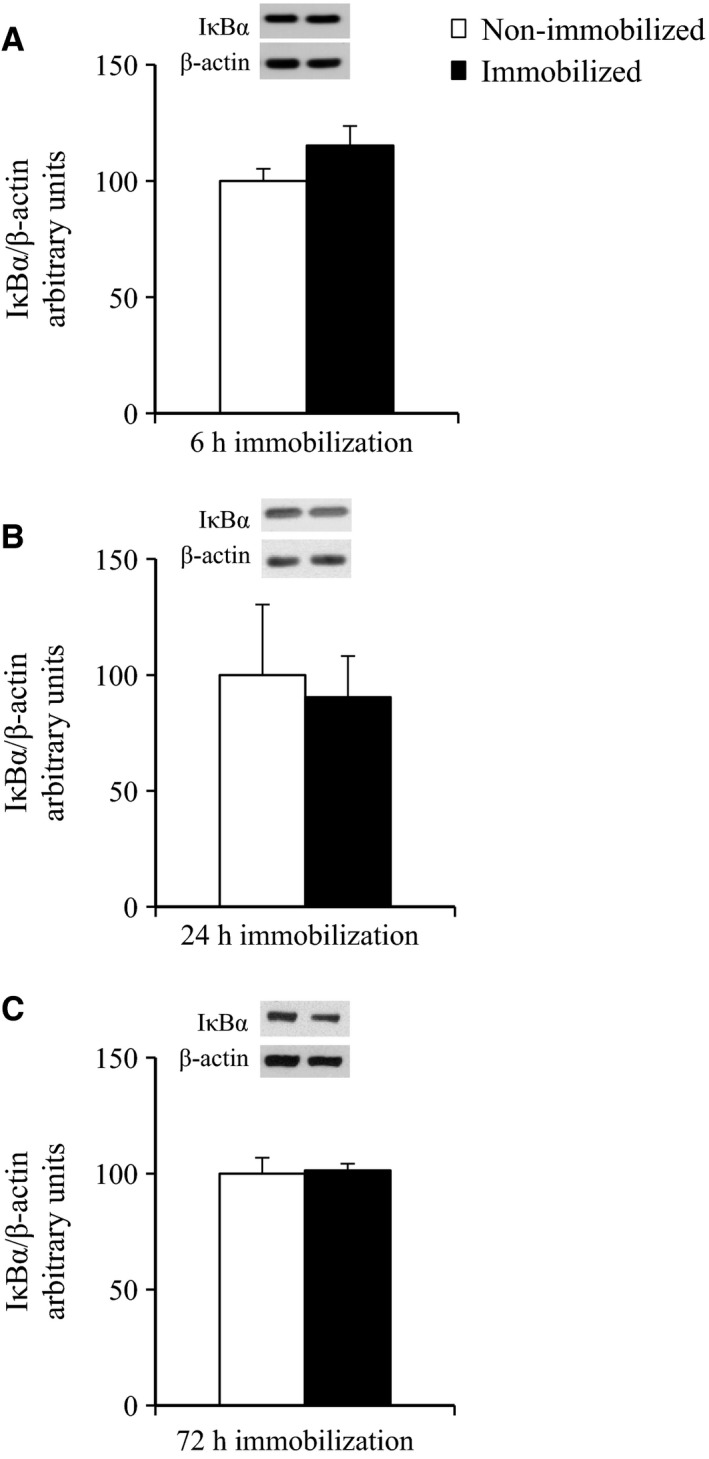
Abundance of I*κ*B*α* protein at the end of 6‐h, 24‐h or 72‐h hindlimb immobilization. Muscles were dissected out and frozen at the end of 6‐h (A), 24‐h (B), or 72‐h (C) unilateral hindlimb immobilization. Muscle lysates were separated with SDS‐PAGE, and blots were analyzed for I*κ*B*α* protein content. Blots were then stripped and analyzed for *β*‐actin. (A) Values are means ± SE (*n* = 10). (B) Values are means ± SE (*n* = 5). (C) Values are means ± SE (*n* = 5).

We estimated the abundance of I*κ*B*α*, which is inversely related to IKK*β* activation and is commonly used as an indicator of activity of the IKK/I*κ*B/NF‐*κ*B pathway. At the end of the 6‐h, 24‐h, or 72‐h immobilization, no significant effect of immobilization on the abundance of I*κ*B*α* was found (Fig. 7A–C).

## Discussion

In this study, the insulin‐stimulated glucose uptake rate was lower for the 6‐h immobilized soleus muscle compared to the contralateral controls (Fig. 1A), suggesting that the loss of postural and ambulatory activity due to hindlimb immobilization rapidly induces muscle insulin resistance. This finding is consistent with a previous study demonstrating that the insulin action of 2DG uptake was decreased in mouse soleus muscle between the third and sixth hours of hindlimb immobilization (Nicholson et al. 1984). We found no significant change in total GLUT4 in the soleus muscles after 6‐h of immobilization (Fig. 1B), showing that the rapid development of insulin resistance in immobilized muscles is not a consequence of decreased total GLUT4 abundance. Therefore, the inactivity‐induced rapid development of insulin resistance is possibly explained by impaired insulin signaling that regulates GLUT4 translocation. This idea is supported by the previous study demonstrating that physical inactivity reduces the ability of insulin to phosphorylate signaling proteins but not total GLUT4 abundance in human skeletal muscle (Mortensen et al. 2014).

Excessive activation of several MAPKs, including JNK and p38 MAPK interferes with normal insulin signaling, leading to the development of insulin resistance in obesity and type 2 diabetes (Blair et al. 1999; Hirosumi et al. 2002; Koistinen et al. 2003; De Alvaro et al. 2004; Sabio et al. 2010; Diamond‐Stanic et al. 2011; Henstridge et al. 2012). Moreover, JNK phosphorylation was reported to be upregulated in rodent soleus muscle in response to prolonged (more than 10 days) physical inactivity (Machida and Booth 2005; Kwon et al. 2015). In this study, shorter duration of inactivity (a 6‐h hindlimb immobilization) resulted in increased phosphorylation of both JNK and p38 MAPK together with the rapid development of insulin resistance for glucose uptake in rat soleus muscle (Figs 1A and 3A, B). In addition, a key finding of this study was that the phosphorylation levels of both JNK and p38 MAPK in immobilized muscle were restored to normal levels by the removal of the plaster cast in conjunction with the restoration of the insulin‐stimulated glucose uptake (Fig. 4A–C). In this context, it is likely that activation of JNK and p38 MAPK is associated with the inactivity‐induced rapid development of insulin resistance for glucose uptake in rat soleus muscle, although our results do not prove causality. Studies using genetic and pharmacologic approaches to manipulate JNK and/or p38 MAPK signaling will help establish the role of this pathway in the rapid induction of insulin resistance in response to short‐duration physical inactivity.

The mechanism responsible for the apparent increase in the phosphorylation of JNK and p38 MAPK signaling in immobilized soleus muscle is not clear. One hypothesis involves the lipid‐induced activation of these signaling pathways. It was previously shown that the JNK and p38 MAPK pathways are activated by lipid infusion or a high‐fat diet (Hirosumi et al. 2002; Prada et al. 2005; Liu and Cao 2009; Bikman et al. 2010). The lipid‐induced activation of the JNK pathway can be triggered by an accumulation of intramyocellular lipid metabolites such as ceramides (Watt et al. 2006; Kewalramani et al. 2010; MohammadTaghvaei et al. 2012; Hassan et al. 2016). We thus hypothesized that accumulation of intramyocellular lipid metabolites are associated with the inactivity‐induced activation of these signaling pathways and with the rapid development of insulin resistance in immobilized muscle. In support of this hypothesis, the abundance of SPT2 (serine palmitoyl transferase‐2), a rate‐limiting enzyme regulating de novo ceramide synthesis from palmitoyl‐CoA, was increased in the soleus muscles after 6‐h of immobilization (Fig. 6A), suggesting that intramyocellular ceramides may be increased in these muscles. Moreover, we found an increased abundance and reduced phosphorylation of ACC in the immobilized muscles (Fig. 5A and B). Since ACC negatively regulates mitochondrial fatty acid *β*‐oxidation through its inhibition of carnitine palmitoyltransferase‐1 (CPT‐1) (Munday 2002), our results (i.e., the increased abundance and reduced phosphorylation of ACC in the immobilized muscles) demonstrate the possibility that immobilization reduces mitochondrial fat oxidation, which may lead to the elevated accumulation of intramyocellular fatty acyl‐CoA such as palmitoyl‐CoA in immobilized muscles. Thus, both reduced mitochondrial fat oxidation and elevated ceramide biosynthesis pathways, and subsequent accumulation of intramyocellular ceramides may contribute to the activation of MAPKs (JNK and p38), although future studies are required to determine whether ceramides are accumulated in immobilized muscle.

It has been postulated that the excessive activation of the IKK/I*κ*B/NF‐*κ*B inflammatory pathway may be an important molecular mechanism responsible for lipid‐induced insulin resistance in skeletal muscle (Kim et al. 2001; Yuan et al. 2001; Itani et al. 2002; Bhatt et al. 2006). In our present study, the abundance of I*κ*B*α* – which is inversely related to IKK and is a commonly used indicator of the activity of the IKK/I*κ*B/NF‐*κ*B pathway – was not decreased in rat soleus muscle within 6 h of hindlimb immobilization (Fig. 7A), although insulin resistance for glucose uptake was rapidly developed in this muscle (Fig. 1). This suggests that activation of the IKK/I*κ*B/NF‐*κ*B pathway is not required for the rapid induction of insulin resistance in 6‐h immobilized muscle. One possible interpretation of our findings is that activation of IKK/I*κ*B/NF‐*κ*B pathway may occur and contribute to the development of insulin resistance, if the period of hindlimb immobilization is extended. However, the abundance of I*κ*B*α* was not changed in immobilized soleus muscle, when we extended the immobilization period from 6 h to 24 h or 72 h (Fig. 7B and C). Therefore, inactivity‐induced insulin resistance in immobilized muscle is unlikely to be mediated through activation of the IKK/I*κ*B/NF‐*κ*B inflammatory pathway, that is consistent with previous study demonstrating that bed rest‐induced (9 days) insulin resistance is not accompanied by increased NF‐*κ*B activity in human skeletal muscle (Friedrichsen et al. 2012).

Thr642 and Ser588 of AS160 are the two primary sites that regulate GLUT4 translocation in response to insulin's activation of Akt (Sano et al. 2003). In this study, we showed for the first time that AS160 phosphorylation in the absence and presence of insulin, measured by specific phosphorylation at sites Thr642 and Ser588, is impaired in the 6‐h immobilized soleus muscle in conjunction with the decrement in the insulin action of glucose uptake (Figs 1A and 2C, D). It is thus very probable that the impaired phosphorylation of AS160 may be involved in the inactivity‐induced rapid development of insulin resistance for glucose uptake in immobilized rat soleus muscle.

We found that, in the immobilized soleus muscle, decreased AS160 (Thr642 and Ser588) phosphorylation in the presence of insulin appeared to be entirely attributable to the smaller baseline values (without insulin) rather than an insulin‐stimulated increase above the basal phosphorylation (Fig. 2C and D). What is a possible mechanism that could link the reduced basal AS160 phosphorylation with decreased insulin‐stimulated glucose uptake in immobilized muscle ? In the basal state, AS160's active Rab GTPase‐activating protein domain is hypothesized to restrain the exocytosis of intracellular GLUT4 storage vesicles (Sano et al. 2003; Sakamoto and Holman 2008). The insulin‐stimulated phosphorylation of AS160 appears to relieve this restraint and allow GLUT4 to be recruited to the cell surface membranes. In this context, decreased basal AS160 phosphorylation may potentiate AS160's inhibitory effect on GLUT4 translocation and render GLUT4 more resistant to a subsequent insulin‐triggered translocation. Therefore, a decrement in basal phosphorylation of AS160 may be important for the decrease in insulin‐stimulated glucose uptake in immobilized soleus muscle.

Muscle contraction activates AMP‐activated protein kinase, resulting in phosphorylation of ACC (Winder and Hardie 1996). In this study, the ACC phosphorylation (a commonly used indicator of AMPK activity) was reduced in the immobilized soleus muscles (Fig. 5B), which is certainly due to the hindlimb immobilization‐induced loss of postural and ambulatory activity. Since AS160 can be phosphorylated independently of Akt by AMPK (Bruss et al. 2005; Kramer et al. 2006a; Geraghty et al. 2007; Treebak et al. 2007, 2010), it may be possible that inactivity‐induced inactivation of AMPK can account for the decrement in basal AS160 phosphorylation and the following decrease in insulin‐stimulated glucose uptake in immobilized soleus muscle. Moreover, previous studies demonstrated that muscle contraction causes a site‐specific phosphorylation of TBC1D1 via AMPK activation (Funai and Cartee 2009; Pehmøller et al. 2009; Frøsig et al. 2010; Vichaiwong et al. 2010), and TBC1D1 phosphorylation on the predicted AMPK phosphorylation site (Ser237) regulates contraction‐stimulated glucose uptake independently of insulin action (Vichaiwong et al. 2010). In this study, the basal phosphorylation of TBC1D1 on AMPK‐targeted phosphomotif (Ser237) and the basal glucose uptake in the absence of insulin were reduced in the immobilized soleus muscles (Figs 1A and 2F). In this context, decreased AMPK activation and the subsequent decrement in TBC1D1 Ser237 phosphorylation can possibly explain the decreased basal glucose uptake in immobilized soleus muscle. Thus, inactivity‐induced inactivation of AMPK may account for reduction in both basal and insulin‐stimulated glucose uptake in immobilized soleus muscle.

In conclusion, we examined two possible hypotheses regarding the mechanism by which inactivity rapidly induces muscle insulin resistance. We found that 6‐h hindlimb immobilization results in the increased phosphorylation of both JNK and p38 MAPK together with the development of insulin resistance for glucose uptake in rat soleus muscle. This finding supports our first hypothesis that the enhanced activation of the proinflammatory/stress pathways, that is, JNK and/or p38 MAPK pathway, is associated with the rapid development of inactivity‐induced insulin resistance in skeletal muscle. Increased abundance of SPT2, a rate‐limiting enzyme regulating ceramide biosynthesis, may contribute to the activation of these pathways. We also found that the basal phosphorylation of AS160 (Thr642 and Ser588) in the absence of insulin is impaired in immobilized soleus muscle, supporting our second hypothesis that the rapid development of muscle insulin resistance linked to inactivity is associated with reduced AS160 phosphorylation.

## Conflict of Interest

None declared.
